# Epigenetic reprogramming of H3K27me3 and DNA methylation during leaf-to-callus transition in peach

**DOI:** 10.1093/hr/uhac132

**Published:** 2022-06-03

**Authors:** Beibei Zheng, Jingjing Liu, Anqi Gao, Xiaomei Chen, Lingling Gao, Liao Liao, Binwen Luo, Collins Otieno Ogutu, Yuepeng Han

**Affiliations:** CAS Key Laboratory of Plant Germplasm Enhancement and Specialty Agriculture, Wuhan Botanical Garden, The Innovative Academy of Seed Design of Chinese Academy of Sciences, Wuhan 430074, China; Hubei Hongshan Laboratory, Wuhan 430070, China; CAS Key Laboratory of Plant Germplasm Enhancement and Specialty Agriculture, Wuhan Botanical Garden, The Innovative Academy of Seed Design of Chinese Academy of Sciences, Wuhan 430074, China; University of Chinese Academy of Sciences, 19A Yuquanlu, Beijing 100049, China; CAS Key Laboratory of Plant Germplasm Enhancement and Specialty Agriculture, Wuhan Botanical Garden, The Innovative Academy of Seed Design of Chinese Academy of Sciences, Wuhan 430074, China; University of Chinese Academy of Sciences, 19A Yuquanlu, Beijing 100049, China; CAS Key Laboratory of Plant Germplasm Enhancement and Specialty Agriculture, Wuhan Botanical Garden, The Innovative Academy of Seed Design of Chinese Academy of Sciences, Wuhan 430074, China; University of Chinese Academy of Sciences, 19A Yuquanlu, Beijing 100049, China; CAS Key Laboratory of Plant Germplasm Enhancement and Specialty Agriculture, Wuhan Botanical Garden, The Innovative Academy of Seed Design of Chinese Academy of Sciences, Wuhan 430074, China; University of Chinese Academy of Sciences, 19A Yuquanlu, Beijing 100049, China; CAS Key Laboratory of Plant Germplasm Enhancement and Specialty Agriculture, Wuhan Botanical Garden, The Innovative Academy of Seed Design of Chinese Academy of Sciences, Wuhan 430074, China; Hubei Hongshan Laboratory, Wuhan 430070, China; CAS Key Laboratory of Plant Germplasm Enhancement and Specialty Agriculture, Wuhan Botanical Garden, The Innovative Academy of Seed Design of Chinese Academy of Sciences, Wuhan 430074, China; University of Chinese Academy of Sciences, 19A Yuquanlu, Beijing 100049, China; CAS Key Laboratory of Plant Germplasm Enhancement and Specialty Agriculture, Wuhan Botanical Garden, The Innovative Academy of Seed Design of Chinese Academy of Sciences, Wuhan 430074, China; Hubei Hongshan Laboratory, Wuhan 430070, China; CAS Key Laboratory of Plant Germplasm Enhancement and Specialty Agriculture, Wuhan Botanical Garden, The Innovative Academy of Seed Design of Chinese Academy of Sciences, Wuhan 430074, China; Hubei Hongshan Laboratory, Wuhan 430070, China

## Abstract

Plant tissues are capable of developing unorganized cell masses termed calluses in response to the appropriate combination of auxin and cytokinin. Revealing the potential epigenetic mechanisms involved in callus development can improve our understanding of the regeneration process of plant cells, which will be beneficial for overcoming regeneration recalcitrance in peach. In this study, we report on single-base resolution mapping of DNA methylation and reprogramming of the pattern of trimethylation of histone H3 at lysine 27 (H3K27me3) at the genome-wide level during the leaf-to-callus transition in peach. Overall, mCG and mCHH were predominant at the genome-wide level and mCG was predominant in genic regions. H3K27me3 deposition was mainly detected in the gene body and at the TSS site, and GAGA repetitive sequences were prone to recruit H3K27me3 modification. H3K27me3 methylation was negatively correlated with gene expression. *In vitro* culture of leaf explants was accompanied by DNA hypomethylation and H3K27me3 demethylation, which could activate auxin- and cytokinin-related regulators to induce callus development. The DNA methylation inhibitor 5-azacytidine could significantly increase callus development, while the H3K27me3 demethylase inhibitor GSK-J4 dramatically reduced callus development. These results demonstrate the roles of DNA methylation and H3K27me3 modification in mediating chromatin status during callus development. Our study provides new insights into the epigenetic mechanisms through which differentiated cells acquire proliferative competence to induce callus development in plants.

## Introduction

Peach (*Prunus persica*) is a member of the genus *Prunus* in the Rosaceae family, which consists of many economically important fruit trees, such as strawberry, pear, apple, and various stone fruit species. Peach is diploid, with a haploid genome size of ~230 million bp (Mb), and has a short juvenile phase of 2–3 years, which could make it an ideal system for genetic research in Rosaceae. However, peach is one of the most recalcitrant species with respect to *in vitro* regeneration. Callus formation is an important step in *Agrobacterium*-mediated transformation [1], and callus can be used in plant biology research and industrial applications [2, 3]. Although various somatic tissues, such as leaf, stem, and calyx, have been successfully used to induce callus in peach, the most commonly used explant is the mature embryo [4, 5]. The main disadvantage of callus induction of seed-derived material is that the seeds are genotypically different from each other and do not represent their parental genotype due to the heterogeneity of fruit crops. To date, few studies have reported on the molecular mechanism(s) underlying callus development in vegetative tissues of peach and of other fruit tree crops.

Callus development from explants relies on the dramatic cell fate transitions from the somatic state to pluripotency [6]. Auxin and cytokinin are two major hormonal regulators of callus induction from explants of somatic tissues during *in vitro* culture [7–9]. To date, remarkable progress has been made towards understanding the molecular mechanism underlying hormone-induced callus development in *Arabidopsis*. Callus development on callus-inducing medium (CIM) is actually through the lateral root-like development pathway, which is regulated by auxin and cytokinin response regulators [10, 11]. Auxin can activate expression of auxin response factors (ARFs) to stabilize the LBD16–bZIP59 complex, leading to both callus development and lateral root formation [12, 13]. The upregulation of WIND1, a wound-induced AP2/ERF transcription factor (TF), can promote callus formation under wound stress, which induces expression of cytokinin biosynthesis and response genes, resulting in accumulation of endogenous cytokinin prior to callus formation [14]. Overexpression of type-B *ARABIDOPSIS RESPONSE REGULATORs* (*ARRs*), which function as TFs in the cytokinin signaling pathway, enhances callus development, and double mutants of *arr1 arr12* and of *arr1 arr10* display defects in callus development [15, 16]. The *CYCD* gene, encoding cyclin D, is a potential target of ARRs and its loss-of-function mutation impairs callus development [15].

Callus induction *in vitro* is associated with wound stress and hormone treatment, which activate many reprogramming regulators that are epigenetically silenced during normal growth [9]. Therefore, much attention has been paid to investigation of the epigenetic regulatory mechanisms underlying wound-/hormone-induced callus development in *Arabidopsis* [17]. The epigenetic mechanisms consist of DNA methylation, histone modification, differential incorporation of histone variants, and non-coding RNAs, all of which influence genome stability and gene transcription [18]. DNA methylation is a form of conserved epigenetic modification that is critical for chromatin stability and gene expression [19]. In plants, DNA methylation occurs in three contexts: CG, CHG, and CHH (where H is A, C or T) [20]. Various DNA methyltransferases have been identified in *Arabidopsis*. METHYLTRANSFERASE 1 (MET1) and CHROMOMETHYLASE 3 (CMT3) are able to maintain CG and CHG methylation, respectively, while CHROMOMETHYLASE 2 (CMT2) and DOMAINS REARRANGED METHYLASE 2 (DRM2) contibute to CHH maintenance [21, 22]. DNA methylation and demethylation vary during developmental stages [23]. Under conditions of tissue culture, changes in DNA methylation are required for re-activation of cell division, leading to callus development [24]. Moreover, *Arabidopsis cmt3* chromomethylase mutations cause significant reduction in CHG context, resulting in a dramatic increase in callus formation capacity [25, 26].

Polycomb repressive complexes (PRCs) are the evolutionarily conserved epigenetic executors in regulating cell fate specification and developmental phase transitions in plants [27]. PRC2 possess the capacity to catalyze the trimethylation of histone H3 at lysine 27 (H3K27me3) [28], and its mutation causes an increase in the competency of somatic embryo induction from vegetative tissues [29, 30]. H3K27me3 is a repressive epigenetic mark associated with genes that are regulated by developmental and environmental cues [31]. Distribution profiles of H3K27me3 are changed when cells convert from a multipotential state to a differentiated state, and vice versa [32, 33]. Genes in the lateral root-like development pathway, which is responsible for callus induction from leaf explants, have been found to decrease H3K27me3 during the leaf-to-callus transition in *Arabidopsis* [32] and rice [34]. Hence, H3K27me3 is crucial for callus development in plants.

H3K27me3 can be demethylated by the JUMONJI DOMAIN CONTAINING 5 (JMJD5) protein, which belongs to the JmjC domain-only subgroup. Removal of the H3K27me3 mark by JMJD5 has been reported at the promoters of core clock genes such as *CIRCADIAN CLOCK ASSOCIATED* (*CCA*) and *LATE ELOGATED HYPOCOTYL* (*LHY*) [35, 36] and the flowering time gene, *FLOWERING LOCUS C* (*FLC*) [37]. Moreover, JMJD5 can activate the *LBD* genes to establish root primordia [38], suggesting its potential roles in callus development. However, to date, our knowledge about the impact of chromatin status, in particular of H3K27me3 modifications, on callus induction and development is very limited in peach and in other fruit trees. In this study we report a genome-wide cytosine methylation map as well as genome-wide reprogramming of the H3K27me3 pattern during the leaf-to-callus transition in peach. The study aims to test whether the epigenetic regulatory mechanisms play a critical role during callus formation in peach and to gain an insight into epigenetic mechanisms underlying callus induction from vegetative tissues. The results of this study will be useful for future development of the plant regeneration system from callus induction in peach.

## Results

### Leaf callus induction and endogenous hormone levels in peach

**Figure 1:**
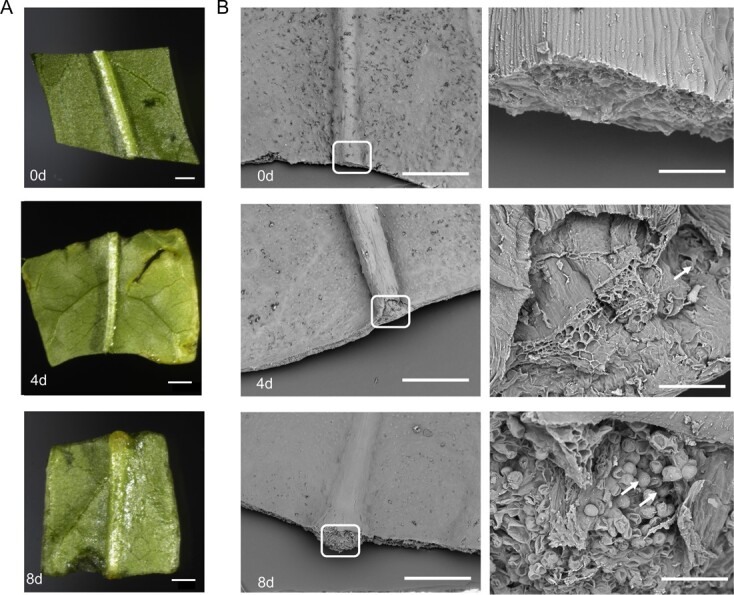
Morphology of leaf explants during callus induction. (A) Leaf explants after 0, 4 and 8 days of culture on CIM. White squares indicate callus cells. (B) SEM analysis of morphology of leaf explants after 0, 4 and 8 days of culture on CIM. White squares indicate callus cells. Images in the right lane are close-ups of the white boxes in the corresponding left lane. Bars represent 2 mm in A and 1 mm and 100 μm in the left and right lanes in B, respectively.

To understand the process of leaf-derived callus formation in peach, fully expanded leaves were cut and placed on callus-inducing medium. After 8 days of culture, clusters of callus structures were formed (Fig. 1A). To investigate the cellular process of callus induction, longitudinal sections of leaf explants at early stages of callus induction were analyzed using an SEM. A few callus cells were induced in the proximal part of the midvein after 4 days of culture, while numerous cells had proliferated 8 days after culture (Fig. 1B). Consistently, callus tissues covered the proximal part of the midvein 8 days after culture, but no calluses were produced around the cut leaf edges. This suggested that peach callus induction is relatively difficult compared with model plants such as *Arabidopsis*, which can easily and rapidly produce callus tissues around cut leaf edges [32].

**Figure 2:**
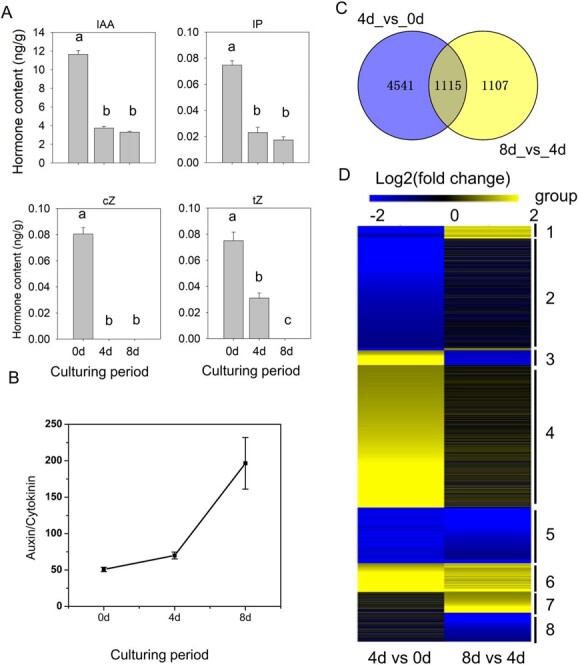
Hormone level determination and RNA-seq analysis of leaf explants during callus induction. (A) Contents of endogenous hormones in leaf explants during the different stages of callus formation. Different lower-case letters indicate statistically significant differences at *P* < 0.05 based on Fisher’s least significant difference test. (B) Ratio of auxin to cytokinin during callus development. Error bars indicate the standard error of three biological replicates. (C) Venn diagrams showing numbers of DEGs during the process of callus induction. (D) Hierarchical clustering of common DEGs in two comparisons. Numbers on the right represent different hierarchical clusters.

Exogenous hormones determine the developmental fate of explants by regulating the distribution of endogenous hormone levels and triggering hormonal response signaling for cell dedifferentiation [9]. Thus, we measured endogenous hormone levels using high-performance liquid chromatography–mass spectrometry (HPLC–MS). The contents of indole-3-acetic acid (IAA), *cis*-zeatin (cZ)-type cytokine, and isopentenyladenine (IP)-type cytokine had significantly decreased after 4 days of culture, and then remained stable between 4 and 8 days (Fig. 2A). The content of *trans*-zeatin (tZ)-type cytokine showed a decreasing trend throughout the culture period. The ratio of auxin to cytokinin was 50.78 in 0-day explants, and was increased to 69.91 and 196.55 in 4-day and 8-day samples, respectively (Fig. 2B).

### Time-course transcriptomes of leaves during callus development in peach

To elucidate the molecular mechanism underlying callus development, transcriptomes of leaf explants at different stages of callus development were compared. We identified 5656 and 2222 DEGs in the 4-day versus 0-day and 8- versus 4-day comparisons, respectively (Fig. 2C). These DEGs were clustered into eight groups based on their temporal expression patterns (Fig. 2D, Supplementary Data Table S1). Group 1 consisted of 203 genes displaying downregulation after 4 days of culture and upregulation after 8 days of culture, while 241 genes from group 3 showed an opposite expression pattern. Groups 2 and 4 contained 1820 and 2318 genes, and displayed down- and upregulation in explants 4 days after culture, respectively, but they both had stable expression between 4 and 8 days. Groups 5 and 6 contained 904 and 470 genes, and showed down- and upregulation, respectively, throughout the culture period. Groups 7 and 8 contained 336 and 471 genes, and they both had stable expression during the first 4 days of culture but showed increasing or decreasing expression after 8 days of culture, respectively. Notably, the majority of DEGs belonged to groups 2 and 4, accounting for 61.2% of the total DEGs. This suggested that most DEGs showed significantly changes in expression after 4 days of culture, but their expression levels were similar between 4 and 8 days of culture.

### Genome-wide DNA methylation landscape during leaf callus development in peach

We screened the annotation of DEGs and found several methylation-related genes, such as two *CMT* genes *PpCMT2* (Prupe.5G024100) and *PpCMT3* (Prupe.6G011600). The expression levels of *PpCMT2* and *PpCMT3* showed downregulation by >2- and 3-fold, respectively (Supplementary Data Fig. S1). This suggested a potential role of DNA methylation in mediating callus development in leaf cultures of peach. As mentioned above, the majority of DEGs dramatically changed in expression after 4 days of culture, but had similar levels of expression between 4 and 8 days of culture. Similarly, hormone accumulation showed dramatic changes after 4 days of culture, but with little change between 4 and 8 days of culture. Therefore, leaf explants at the beginning (0) and after 4 days of culture were chosen to investigate genome-wide profiles of DNA methylation using whole-genome bisulfite sequencing (WGBS). The raw read numbers for the bisulfite sequencing libraries varied from 37 566 294 to 48 916 272. After filtering, 36 800 735–47 952 059 clean reads with total size ranging from 10.06 to 13.12 Gb were obtained for each library. The clean reads for each library covered the peach reference genome at ~50× depth. On average, the conversion rate of bisulfite-treated genomic DNA was 99.5%. The average percentages of methylated cytosine in the sequence contents of CG, CHG, and CHH were 49.6, 27.3, and 7.1%, respectively (Supplementary Data Table S2).

**Figure 3:**
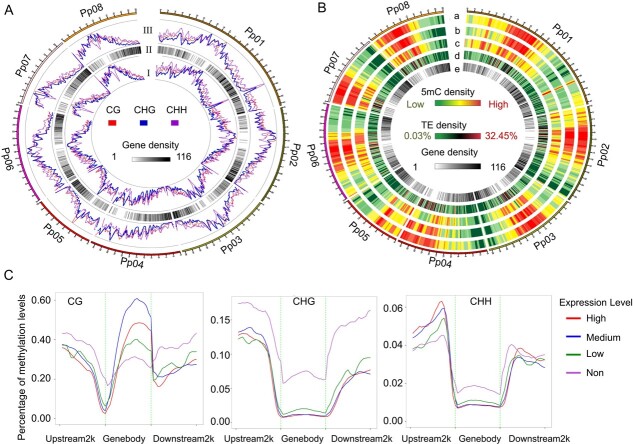
Peach epigenome of leaf explants during callus development. (A) Circos plot displaying the level (I) and density (III) of methylated cytosines (mCs) in the CG, CHG, and CHH contexts, and gene density (II) in each peach chromosome. The contexts of CG, CHG, and CHH are shown as red, blue, and purple lines, respectively. (B) Circos plots of peach chromosomes. Track order: (a) density plot of mC in CG methylation type; (b) density plot of mC in CHG methylation type; (c) density plot of mC in CHH methylation type; (d) TE density; (e) density of genes in each chromosome. (C) Distribution of methylation context and levels in gene body regions partitioned by gene expression levels. Genes with FPKM value <1 were considered non-expressed (Non); genes with FPKM value from 1 to 6 were considered Low; genes with FPKM value from 6 to 45 were considered Medium and genes with FPKM value >45 were considered High.

Overall, the distribution of the three different types of methylated cytosine was similar between the 0- and 4-day explants. The proportions of mCG, mCHG and mCHH to total mC sites were 37, 37, and 26%, respectively (Supplementary Data Fig. S2). The gene-rich regions showed low levels of DNA methylation, while the transposable element (TE)-rich regions had high levels of DNA methylation in 4-day samples (Fig. 3A and B), and similar distribution profiles were also observed in 0-day samples (Supplementary Data Fig. S3). Subsequently, DNA methylation levels were evaluated in gene body and flanking regions that contained 2 kb downstream and upstream of the transcription end site (TES) and transcription start site (TSS), respectively (Supplementary Data Fig. S4). The CG context had the highest frequency of methylation in the gene body, while both CHG and CHH displayed the highest frequency of methylation in the 2-kb upstream region. Within the gene body, the CG context had higher methylation frequency in introns and exons than both CHG and CHH. The lowest methylation levels at CG, CHG, and CHH sites were observed within regions adjacent to TSS and TES.

The effect of DNA methylation in the gene body and flanking regions on gene transcription was investigated (Fig. 3C). Genes were divided into four sets with no, low, medium, or high expression. As expected, highly expressed genes displayed low methylation levels of mCHG in most genic regions, while an opposite pattern was observed for the non-expressed genes. However, a complicated methylation pattern was detected for both CG and CHH. The non-expressed genes showed a high methylation level of CG in the 2-kb upstream and 2-kb downstream regions, especially at the TSS and TES sites. By contrast, the medium- and high-expressed genes displayed high methylation levels of CG in the gene body. Notably, the methylation levels of CHH in the 2-kb upstream region were positively correlated with gene expression levels, while a negative correlation occurred in the gene body. Gene expression levels showed no association with the CHH methylation levels in the 2-kb downstream region and around the TSS and TES sites. The positive association of the mCHH methylation level in the 2-kb upstream region with gene expression is consistent with previous reports in rice [55] and apple [41].

**Figure 4:**
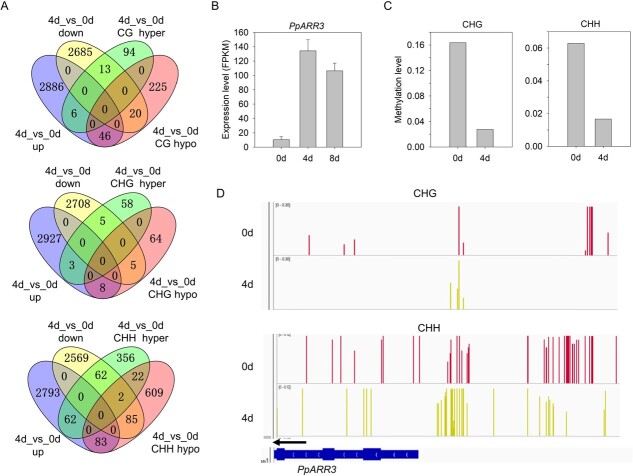
Integrated methylome and transcriptome analysis. (A) Venn diagrams showing the numbers of down- and upregulated DEGs as well as hyper- and hypo-DMRs containing any of mCG, mCHG, and mCHH located in the promoter regions of the DEGs. (B) Expression profiles of *PpARR3* at three stages of callus development. (C) CHG and CHH methylation difference of *PpARR3* at two stages of callus development. (D) IGV software depicts CHG and CHH hypomethylation in the promoter region of *PpARR3* at two stages of callus development. Arrows indicate the transcription direction.

As differentially methylated regions (DMRs) are potential functional regions involved in gene transcriptional regulation, DMRs were investigated between 0-day and 4-day explants (Supplementary Data Table S3; Supplementary Data Fig. S5A). We identified 2188 hypomethylated and 1375 hypermethylated DMRs. All DMRs could be divided into three types: mCG-DMRs, mCHG-DMRs, and mCHH-DMRs. Overall, mCHH-DMRs were most abundant, followed by mCG-DMRs and mCHG-DMRs. During leaf callus development, the number of hypomethylated CG- and CHH-DMRs was higher than that of hypermethylated CG- and CHH-DMRs, while the number of hypomethylated CHG-DMRs was lower than that of hypermethylated CHG-DMRs in TE and other regions. In the promoter sequences, the number of hypomethylated CHG-DMRs was higher than the number of hypermethylated CHG-DMRs (Supplementary Data Fig. S5B and C). All the 3563 DMRs occurred within 727 gene bodies and 1730 gene promoters. Most of the gene bodies and gene promoters contained only a single type of DMR (Supplementary Data Fig. S5D). A negative correlation between gene expression and DNA methylation was detected for these DMR-related genes, with either promoter containing hypo-mCG/mCHG or gene body containing hypo-mCHG/mCHH or hyper-mCHG (Supplementary Data Fig. S6).

Since DNA methylation in the promoter region has a negative impact on gene expression, we focused on DMRs in gene promoters. A total of 398 DMRs were identified in the promoter proximal to DEGs. Of the 398 DMRs in promoters, 80 were hypermethylated DMRs associated with downregulated DEGs, and 137 were hypomethylated DMRs associated with upregulated DEGs (Fig. 4A). Among the 80 hypermethylated DRMs in promoters, 13, 5, and 62 belonged to mCG, mCHG and mCHH, respectively. Likewise, 46, 8, and 83 out of the 137 hypomethylated DMRs in promoters belonged to mCG, mCHG, and mCHH, respectively. Notably, 19 out of these 217 DMRs in promoters were identified to be proximal to TFs deposited in the PlantTFDB database (http://planttfdb.cbi.pku.edu.cn/) (Supplementary Data Table S3). Interestingly, one cytokinin response TF, *PpARR3* (Prupe.1G494200), is an *ARR* homolog*.* In the *PpARR3* promoter, DNA methylation level was dramatically decreased after 4 days of culture, which is consistent with the upregulation of its expression by >4-fold (Fig. 4B–D).

### Genome-wide H3K27me3 modification landscape during leaf callus development in peach

**Figure 5:**
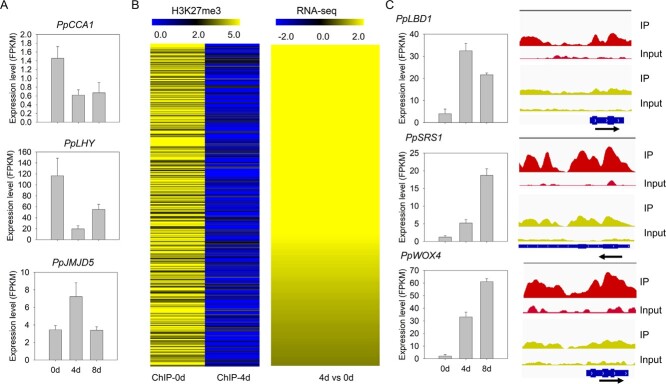
Genome-wide profiling of H3K27me3 methylation and gene expression during peach callus development. (A) Expression levels of *PpCCA1*, *PpLHY*, and *PpJMJD5* at three stages of callus formation. (B) Hierarchical clustering of altered H3K27me3 deposition regions and their corresponding DEGs. (C) Expression profiles (left) and H3K27me3 depositions (right) of *PpLBD1*, *PpSRS1*, and *PpWOX4*. Arrows indicate the transcription direction.

The JMJD5 protein, a well-studied histone demethylase in *Arabidopsis*, is a member of the JmjC-domain-only subgroup that removes the H3K27me3 mark [35]. The *JMJD5* gene and its repressors *CCA* and *LHY* form a feedback circuit to regulate circadian oscillation. Transcriptome analysis revealed that *PpJMJD5* expression was upregulated by >2-fold after 4 days of culture, while the expression levels of *PpCCA1* and *PpLHY* were downregulated by 2.5- and 6.3-fold, respectively (Fig. 5A). This suggested a potential role of H3K27me3 modification in mediating leaf callus development in peach. Hence, ChIP-seq was conducted to investigate the genome-wide H3K27me3 profile during leaf callus development. Overall, the peaks of H3K27me3 at the whole-genome level were mainly enriched in intergenic regions (Supplementary Data Fig. S7A). For the gene regions in peach, the highest levels of H3K27me3 modification were mainly observed either in the gene body or around the TSS, while few peaks were found in flanking regions 3 kb upstream and downstream of the TSS and TES, respectively (Supplementary Data Fig. S7B and C). In addition, TA, GA, and GAGA repetitive sequences were prone to recruit H3K27me3 modification (Supplementary Data Fig. S7D).

A total of 5748 and 2430 genes containing H3K27me3 marks were identified for the 0-day and 4-day explants, respectively. A total of 5114 genes displayed a difference in H3K27me3 enrichment. Among these genes, 4861 showed a decrease in H3K27me3 modification (Supplementary Data Table S4), while 253 genes displayed increased enrichment of H3K27me3. This suggested a decrease in H3K27me3 modification during leaf callus development. Of the 253 H3K27me3-increased genes, 62 showed a significant difference in expression after 4 days of culture (Supplementary Data Table S4). However, these 62 genes showed no association with callus formation based on the peach reference genome annotation, and they were not included in further analyses. Of the 4861 H3K27me3-decreased genes, 642 belonged to TFs, accounting for 36.2% of total TFs in the peach genome that were deposited in the PlantTFDB database. These H3K27me3-decreased TFs mainly belonged to the *AP2*, *bHLH*, *MYB*, *NAC*, *WRKY*, *WOX*, *TCP MADS*, *GRAS*, *Orphans*, and *LOB* families (Supplementary Data Table S5).

**Figure 6:**
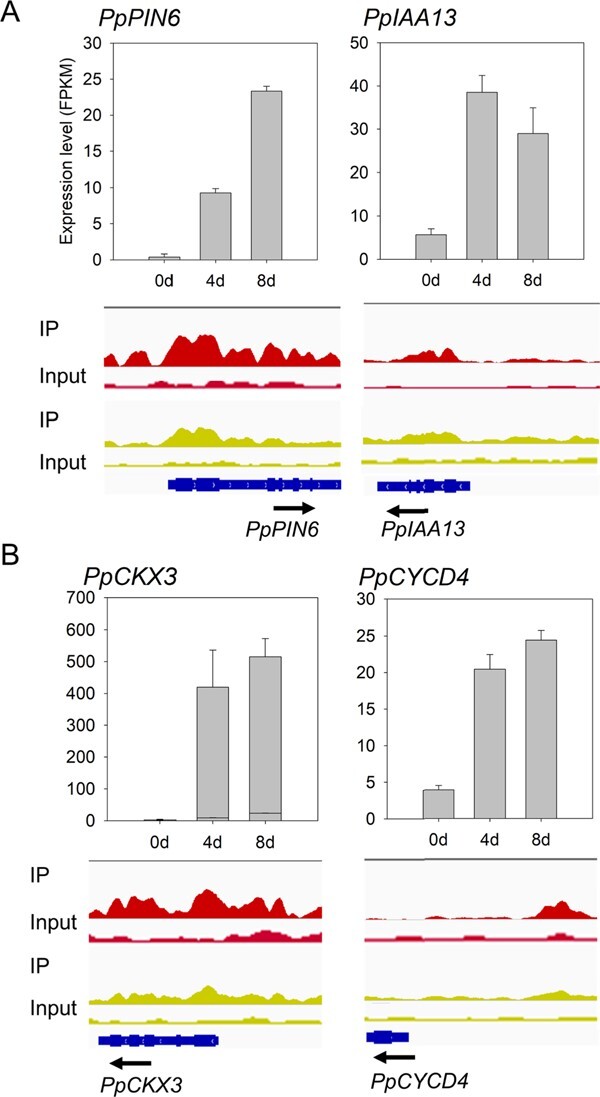
Expression and methylation changes in auxin- and cytokinin-related genes during callus development in peach. (A) Expression profiles (top) and H3K27me3 depositions (bottom) of *PpPIN6* and *PpIAA13* involved in auxin transport and response. (B) Expression profiles (top) and H3K27me3 deposition (bottom) of *PpCKX3* and *PpCYCD4* involved in cytokinin degradation and response.

GO annotation revealed that the 4861 H3K27me3-decreased genes were mainly involved in cytokinin metabolic process, receptor binding, and regulation of gene expression (Supplementary Data Fig. S8). Of these 4861 genes, 1188 showed a significant difference in expression after 4 days of culture (Supplementary Data Table S4). Since the enrichment levels of H3K27me3 have a negative impact on gene expression in peach [56], we focused on a set of 591 genes that exhibited decreased enrichment of H3K27me3 and upregulation after 4 days of culture (Fig. 5B and C). Screening of the 591 genes revealed two relevant to auxin, *PpPIN6* (Prupe.8G018200) encoding an auxin efflux carrier (PIN) and *PpIAA13* (Prupe.7G247500) encoding a putative auxin-responsive Aux/IAA protein (Fig. 6A). Moreover, a cytokinin oxidase/dehydrogenase (CKX) genes, *PpCKX3* (Prupe.7G208400) as well as a cytokinin-induced gene *PpCYCD4* (Prupe.6G196300), were found (Fig. 6B). All these auxin- and cytokinin-related genes showed decreased enrichment of H3K27me3 and upregulation after 4 days of culture.

Of the 591 genes, 66 belong to TFs, and some of them are associated with meristematic differentiation. For example, as shown in Fig. 5 and Supplementary Data Fig. S9, three TFs, *PpLBD1* (Prupe.6G093900), *PpLBD38* (Prupe.8G142100), and *PpLOB* (Prupe.3G290800), belong to the Lateral Organ Boundaries (LOB) family controlling lateral organ fate. Two TFs, *PpSRS1* (Prupe.7G163600) and *PpLRP1* (Prupe.2G036500), are members of the Shi-related sequence (SRS) family involved in lateral root primordium. One *WUSCHEL* (*WUS*)-related TF, *PpWOX4* (Prupe.1G432100), belongs to the HB family related to callus development. Five *NAC* TFs, *PpNAC39* (Prupe.1G454900), *PpNAC58* (Prupe.5G221600), *PpNAC43* (Prupe.8G074800), *PpNAC30* (Prupe.8G058100), and *PpNAC25* (Prupe.4G040900), are homologs of *Arabidopsis CUP-SHAPED COTYLEDON* (*CUC*) genes regulating shoot apical meristem (SAM) formation and auxin-mediated lateral root formation [57]. These results indicated that variation in H3K27me3 deposition could contribute to changes in the expression patterns of TFs involved in callus development.

### Decreased methylation has a positive effect on leaf callus induction in peach

**Figure 7:**
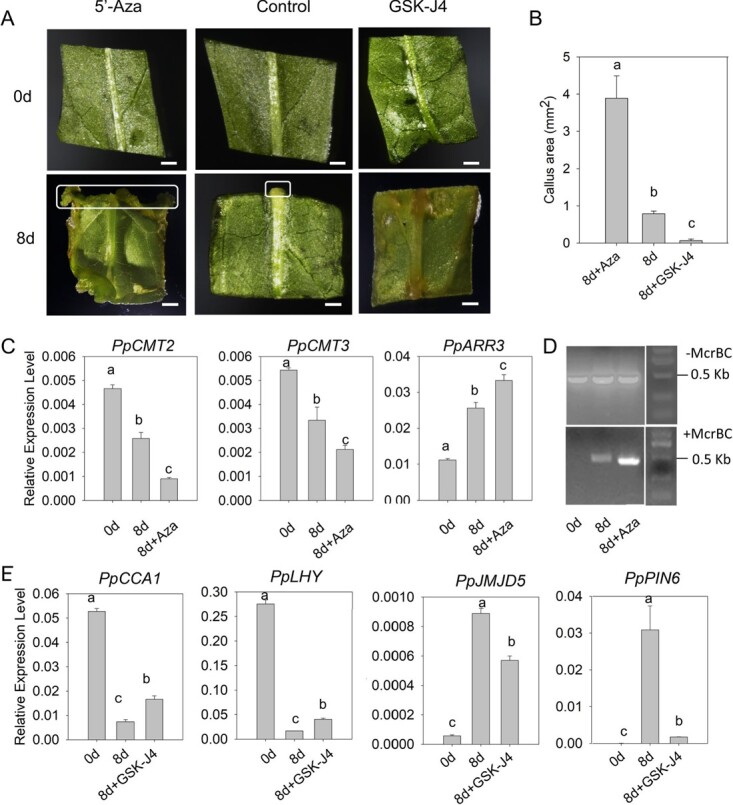
Effect of DNA methylation inhibitor 5′-azacytidine and H3K27me3 demethylase inhibitor GSK-J4 on peach callus formation. (A) *In vitro* callus induction on CIM supplied with 5′-azacytidine and GSK-J4. White squares indicate callus cells. Scale bars = 2 mm. (B) Statistical analysis of callus areas. Eight explants were measured for each treatment. (C) Expression profiles of *PpCMT2*, *PpCMT3*, and *PpARR3*. (D) Detection of DNA methylation status in the promoter region of *PpARR3* by McrBC–PCR analysis. (E) Expression of genes involved in H3K27me3 methylation. Different lowercase letters in (B), (C) and (E) indicate a statistically significant difference at *P* < 0.05 based on Fisher’s least significant difference test.

To validate the impact of DNA methylation on leaf callus development, the DNA methylation inhibitor 5-azacytidine was added to callus-inducing medium. After 8 days of culture, abundant calli were observed around both the proximal part of the midvein and the cut edges of leaf explants incubated on CIM supplied with 5-azacytidine (Fig. 7A). However, callus was only formed around the proximal part of the midvein of leaf explants incubated on CIM without 5-azacytidine. The size of total callus areas in leaf explants with 5-azacytidine treatment was significantly greater compared with that in leaf explants with mock treatment (Fig. 7B). Expression of two genes controlling DNA methylation, *PpCMT2* and *PpCMT3*, was significantly downregulated (Fig. 7C), while the DNA methylation-sensitive gene *PpARR3* was upregulated upon treatment with 5-azacytidine. Consistently, McrBC–PCR analysis showed that the promoter region of *PpARR3* was hypomethylated in leaf explants after 8 days of treatment with 5-azacytidine (Fig. 7D). These results indicated a positive effect of DNA hypomethylation on leaf callus induction in peach.

**Figure 8:**
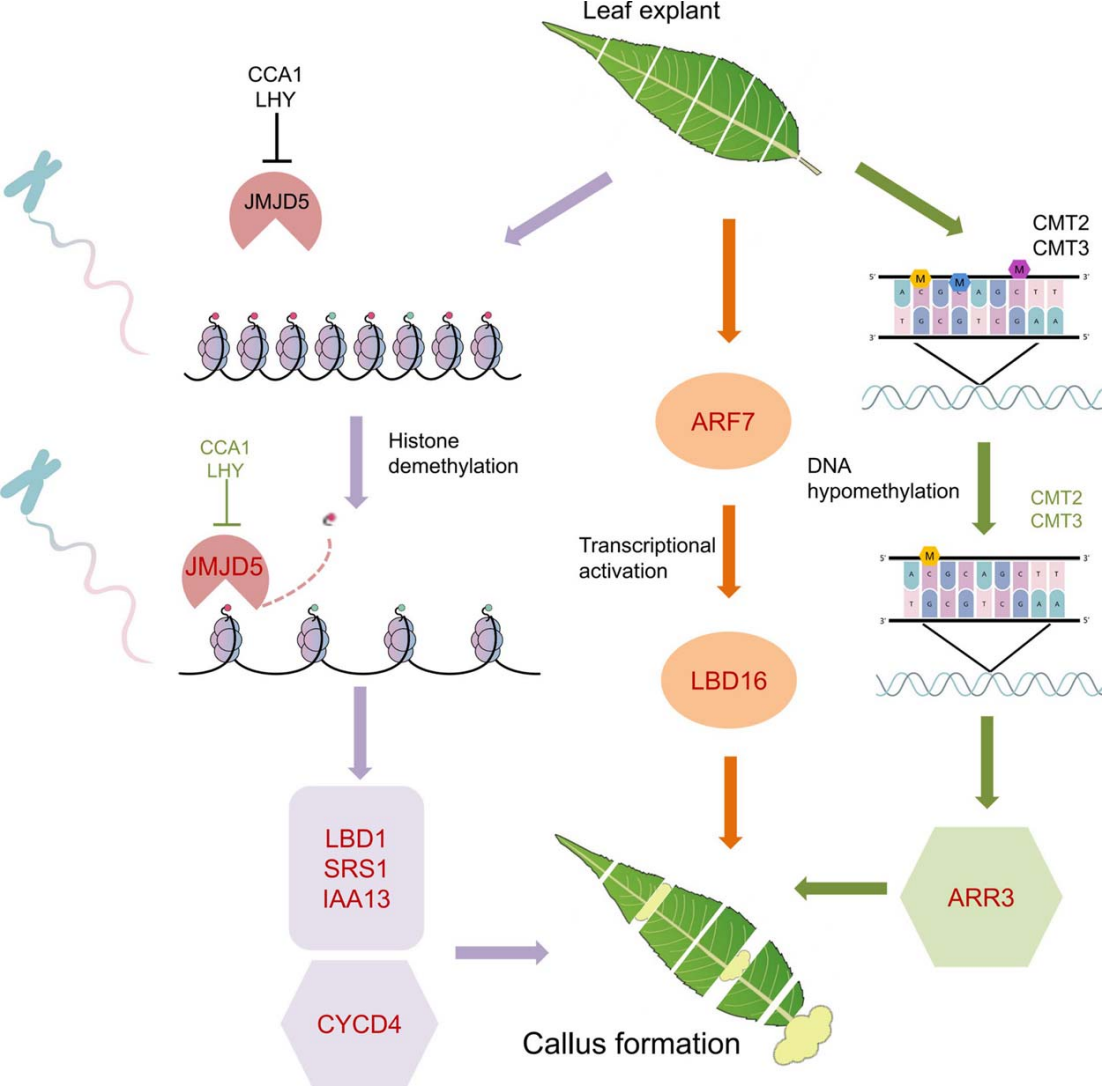
A proposed model for epigenetic modification during callus development. DNA methylation-dependent and H3K27me3-dependent callus induction pathways are indicated by dark green and purple arrows, respectively, whereas, the DNA methylation- and H3K27me3-independent pathway is indicated by dark orange arrows. Auxin- and cytokinin-related genes are marked with rectangular and hexagonal background shapes, respectively. Significantly up- or downregulated genes are highlighted in red and green colors, respectively, while their original expression at the beginning of culture is shown in black.

Likewise, to validate the impact of histone methylation on leaf callus development, the H3K27me3 demethylase inhibitor GSK-J4 was added to CIM. After 8 days of culture, no callus development was observed around either the proximal part of the midvein or the cut edges of leaf explants incubated on CIM supplied with GSK-J4 (Fig. 7A). The size of total callus areas in GSK-J4 treated leaf explants was significantly smaller compared with that in mock-treated leaf explants (Fig. 7B). Expression of *PpJMJD5* was significantly downregulated, while its repressors *PpLHY* and *PpCCA1* showed increased expression upon treatment with GSK-J4 (Fig. 7E). Significant downregulation was also detected for *PpPIN6*. These results demonstrated a potential role of H3K27me3 demethylation in leaf callus induction in peach.

## Discussion

Callus can be induced *in vitro* by exogenous application of plant hormones, and its developmental fate is determined by the balance between auxin and cytokinin. A high ratio of auxin to cytokine promotes root formation, while a low ratio facilitates shoot development [8]. In this study, the ratio of auxin to cytokine increased during the process of callus development. The lateral root-related *LBD* TFs showed a significant decrease in the H3K27me3 level and were thus upregulated during the leaf-to-callus transition. However, *Prupe.7G146900*, a homolog of the *Arabidopsis SAW*1 gene controlling leaf development, showed no significant change in either H3K27me3 or DNA methylation levels, but its expression was significantly downregulated after 4 days of culture. These results clearly indicate that CIM-induced peach leaf callus occurs through a lateral root development pathway, in agreement with previous studies [17, 58]. Additionally, callus induction was only observed in the proximal part of the midvein after 8 days of culture. This finding is consistent with a previous report that callus induction on CIM is prone to occur in the pericycle or pericycle-like cells adjacent to the xylem poles through formative or asymmetric divisions [10, 59].

Cell fate transition during callus development requires global reprogramming of DNA methylation. Here, our results showed that the DNA methylation level in the peach genome is lower than those in crops with relatively larger genome sizes, such as tomato [60], apple [41], and cotton [61], but higher than that in *Arabidopsis*, with a relatively smaller genome size [54]. This confirms the previous finding that DNA methylation levels are positively correlated with genome size [62]. Notably, DNA methylation at the genome-wide level preferentially occurred in the CG and CHH contexts in peach, which is in contrast to the reported predominant mC in the CG context in *Arabidopsis* [55] and in the CHH context in apple [41], tomato [60], and cotton [61]. In terms of the gene level, DNA methylation predominantly occurred in the CG context in the promoter and gene body regions in peach, which is consistent with the recent finding of CG methylation instead of the CHG or CHH methylation predominant in genic regions in peach fruit [63]. Additionally, DNA methylation levels were positively correlated with TE density, but had a negative correlation with gene density in peach, which supports previous reports in other plant species [62, 64, 65].

Besides maintaining genome stability, DNA methylation has an effect on gene expression. In this study, gene expression levels negatively correlated with the frequency of DNA methylation in the CG context of promoter regions, but showed a positive correlation with the frequency of DNA methylation within the gene body, in agreement with a previous report in rice [66]. Interestingly, our study revealed that the DNA methylation inhibitor 5-azacytidine could significantly increase callus development. This suggests an important role of DNA hypomethylation in callus development. It is well known that the regulatory role of 5-azacytidine in DNA methylation is attributed to its inhibitory effect on DNA methyltransferases [67]. Our results showed that exogenous hormone application resulted in a decrease in the expression of genes encoding DNA methyltransferases during the leaf-to-callus transition in peach. Hence, the influence of exogenous hormones on epigenetic modifications could be partially due to changes in the expression of DNA methyltransferase genes. The regulaotry role of genes encoding DNA methyltransferases in callus formation has also been reported in *Arabidop*sis, in which mutants of *cmt3* [26] and *drm1/drm2* [69] show a positive effect on callus formation. However, the DNA methyltransferase genes *CMT2* and *CMT3,* homologs of *PpCMT2/3* in this study, are upregulated during the process of callus formation in strawberry [68]. Thus, the role of DNA methyltransferase genes in callus formation is not conserved across plant species. Additionally, 5-azacytidine showed a negative impact on leaf culture in the micromolar concentration range (data not shown). The inhibitory effect of 5-azacytidine on callus formation in the micromolar concentration range has also been reported in wheat [67]. 5-Azacytidine in the nanomolar concentration range shows a positive effect on callus formation in wheat, which is consistent with our finding in this study.

Unlike DNA methylation, H3K27me3 modification was mainly detected in genic regions, including the gene body and the TSS site, but seldom detected in flanking regions during the process of callus induction in peach. This is in agreement with findings previously reported in *Arabidopsis* [32]. The GAGA factor-binding motif was enriched in H3K27me3 ChIP-seq data in the present study, which is consistent with previous reports in *Arabidopsis* [70], rice [71], and peach [56]. The genome-scale H3K27me3 pattern is determined by the balance between histone methylation and histone demethylation. Overexpression of *JMJD5* regulating diurnal histone modification of clock genes results in a reduction of H3K27me3 deposition [37]. In our study, the expression level of *JMJD5* was significantly upregulated after 4 days of culture, and was enhanced by simultaneous downregulation of its repressors *CCA1* and *LHY*, leading to a dramatic reduction in H3K27me3 deposition. This indicates that *JMJD5* could serve as an important node for mediating callus development. Expectedly, callus was dramatically reduced by exogenous application of the H3K27me3 histone demethylase inhibitor GSK-J4, which significantly repressed the expression of *PpJMJD5*. This finding is consistent with the report that GSK-J4 can affect the proliferation and apoptosis of various cancer cells through repressing the expression of *JMJD3* (a histone H3K27 demethylase) in animals and humans [72–74]. It is worth noting that H3K27me3 deposition may be transiently diluted during cell division, but the H3K27me3 level can be quickly restored to normal levels [69]. Thus, this dilution by DNA replication is unlikely to be responsible for H3K27me3 demethylation during peach callus formation.

Our results showed that decreased H3K27me3 deposition occurred in TFs associated with plant regeneration, such as SAM-related NACs and LBD/WOX TFs, leading to an increase in their expression. This negative correlation indicated that H3K27me3 methylation participates in the regulation of callus development at the transcriptional level in peach. Additionally, H3K27me3 has been reported to participate in chromatin condensation and transcription silencing [75, 76]. Our study shows that peach callus development is accompanied by H3K27me3 demethylation at the genome-wide level. This finding is consistent with the fact that cellular dedifferentiation and proliferation during plant tissue culture is associated with large-scale chromatin decondensation [77, 78].

Antagonistic function between H3K27me3 and DNA methylation has been reported in imprinted loci in *Arabidopsis* [79]. However, DNA and H3K27me3 methylation are likely to be mutually reinforced in a set of functional genes in rice [71]. Here, 153 DEGs were identified to display significant changes in both DNA methylation and H3K27me3 deposition (Supplementary Data Table S4). Among the 153 DEGs, one is *PpARR3*, which is homologous to *Arabidopsis AtARR*s involved in callus formation. *PpARR3* displayed a decrease in both DNA methylation and H3K27me3 modification during the process of callus formation. Thus, reduced H3K27me3 and DNA hypomethylation seem to reinforce the transcription of *PpARR3*, suggesting cooperation between H3K27me3 and DNA methylation during peach callus formation.

Although auxin and cytokinin are commonly used to induce callus from explants *in vitro*, our knowledge of the molecular mechanisms underlying callus development is limited in plants. Comparative transcriptome analysis revealed that the auxin response genes *PpARF7* and *PpIAA13* and their downstream genes *PpLBD16*, *PpLBD1*, and *Pp*S*RS1* were upregulated during callus development. Likewise, upregulation was also observed for the cytokinin response gene *PpARR3* and its downstream gene *PpCYCD4*. As mentioned above, the DNA methyltransferase genes *CMT2* and *CMT3* were downregulated due to exogenous hormones in CIM, and H3K27me3 and DNA methylation were reduced during the process of callus formation. This is likely responsible for the activation of *PpARR3*, although the concentration of endogenous cytokinin showed a decrease during the process of callus formation. Of these genes, *PpCYCD4* is one of the core cell cycle regulators. By contrast, a cell cycle inhibitor, *PpKRP*, was downregulated during peach callus development, consistent with the previous finding that exogenous auxin and cytokinin can induce cell cycle re-entry from G1 to S phase, leading to proliferation and callus development [78]. Methylation analysis further revealed that three upregulated genes, *PpLBD1*, *Pp*S*RS1*, and *PpIAA13*, showed decreased H3K27me3 deposition, while two upregulated genes, *PpARR3* and *PpCYCD4*, displayed DNA hypomethylation and H3K27me3 demethylation, respectively, during callus development. These results suggest the roles of DNA and histone methylation in mediating callus development in peach. Notably, upregulation of *PpLBD16* and *PpARF7* showed no association with either DNA hypomethylation or H3K27me3 demethylation. A recent study demonstrated that cellular dedifferentiation is facilitated by the H3K36me3 deposition that is catalyzed by ARABIDOPSIS TRITHORAX-RELATED 2 (ATXR2) in the promoters of *LBD* genes in *Arabidopsis* [80]. Therefore, it cannot be excluded that upregulation of *PpLBD16* and *PpARF7* might be attributed to other types of histone modifications.

Based on all the above results, we propose a model for epigenetic mechanisms underlying leaf callus development in peach (Fig. 8). Downregulation of *PpCMTs* causes a decrease in DNA methylation, while upregulation of *PpJMJD5* as well as downregulation of its repressors *PpCCA1* and *PpLHY* results in decreased H3K27me3 deposition. H3K27me3 demethylation results in activation of auxin-related regulators, while both DNA hypomethylation and H3K27me3 demethylation could activate cytokinin-related regulators, resulting in callus induction. However, two callus development regulators, *PpLBD16* and *PpARF7*, show no association with either DNA hypomethylation or H3K27me3 demethylation, which may correspond to genetic regulation of callus formation. These results will improve our knowledge of reprograming of cellular differentiation to regain the proliferative competence required for callus development in plants.

## Materials and methods

### Plant materials and callus induction

Leaf samples were collected from *in vitro*-grown proliferating shoot cultures of peach cv. ‘Shengli’ (*P. persica*), which were maintained on Murashige and Skoog (MS) medium with 1% sucrose at 25°C and humidity of 60–70% under a 16-hours light/8-hours dark cycle. For callus induction, fully expanded leaves were cut into small sections (1 cm × 1 cm) using a disposable knife, and cultured in CIM in darkness at 25°C and 60–70% humidity. The CIM was MS basal medium with vitamins (M519, PhytoTech, USA) (4.43 g/L), 2,4-dichlorophenoxyacetic acid (2 mg/L), sucrose (30 g/L), agar (8 g/L), and 6-Benzylaminopurine (BA) (0.5 mg/L), pH 5.8.

### Measurement of endogenous plant hormones

Approximately 120 mg of fresh callus was frozen and ground into powder in liquid nitrogen, and extracted with a mixture of 80:20 methanol/water (v/v) at 4°C. Endogenous hormones were extracted and quantified by Metware Biotechnology Co., Ltd (Wuhan, China) following a previous report [39]. Each treatment consisted of three biological replicates.

### Analysis by scanning electron microscopy

Callus formation from leaf explants was examined using a scanning electron microscope (SEM). The leaf explants were collected and dried with a vacuum freeze-dryer. All samples were prepared and viewed with an SEM (Hitachi TM3030) according to a previous study [40].

### Construction of bisulfite sequencing libraries and analysis of differentially methylated regions

Leaf explants at different periods of culture were collected and ground into powder in liquid nitrogen. Approximately 100 mg of powder was subjected to DNA extraction using the EasyPure Plant Genomic DNA Kit (EE111–01). Bisulfite sequencing libraries were prepared with the EZ DNA Methylation-Gold™ Kit (Zymo Research, Irvine, CA, USA) following a previous report [41]. Library sequencing was conducted on the Illumina Hiseq 2500 platform with sequencing depths >50×. Differentially methylated region (DMRs) were analyzed with DSS software (version DSS_2.12.0) [42], and regions with at least four methylated cytosines were considered as DMRs [41]. The Circos plot was drawn using R software (version 3.5.3). Each treatment contained three biological replicates.

McrBC–PCR analysis was performed according to a previous study [43] and primer sequences were listed in Supplementary Data Table S6. Briefly, ~1 μg of genomic DNA was digested with 20 units of restriction endonuclease (NEB, cat. no. M0272S). The McrBC-digested template along with a mock digested template was subjected to PCR analysis.

### Analysis of chromatin immunoprecipitation sequencing

Chromatin immunoprecipitation (ChIP) was carried out with the Universal Plant ChIP-seq kit (Diagenode, stock number C01010152) following a previous study [44]. Briefly, ~5 g of leaf explant was vacuum-infiltrated five times for 15 minutes in cross-linking solution at room temperature. After stopping treatment, the explants were ground to fine powder in liquid nitrogen. The extracted chromatin was sheared by sonication into fragments ranging from 200 to 500 bp. The sonication was carried out with 10 cycles of 30 seconds on/off using Bioruptor™ UCD 200 (Diagenode, Sparta, NJ). Chromatin-associated DNA was immunoprecipitated using mouse H3K27me3 monoclonal antibody (Abcam, stock number ab6002).

DNA sequencing was carried out using an Illumina HiSeq2000 platform at Beijing Genomics Institute (BGI, Shenzhen, China). Clean reads with a minimum quality score of Phred20 or Q20 were aligned against the reference genome of peach (https://www.rosaceae.org/organism/24333) using Burrows-Wheeler Aligner (version 0.7.10-r789) [45]. ChIP sequencing (ChIP-seq) peak calling was conducted using MACS2 and visualization was performed using Integrative Genomics Viewer [46]. The ChIP-seq signal was normalized based on the total number of sequenced fragments per sample. Peak annotation was carried out using the HOMER annotatePeaks.pl script. Three biological replicates were performed for enrichment, and the enriched DNA samples were mixed to prepare a sequencing library. Sequencing was conducted at 50× depth.

### RNA extraction, transcriptome analysis and real-time PCR

Plant materials for transcriptome analysis were collected in a way similar to the ways used for methylome and ChIP-seq analyses. RNA extraction and sequencing were conducted following a previous report [41]. Each treatment consisted of three biological replicates. The adapter and low-quality bases were deleted from raw data and subsequently aligned against the reference genome of peach using TopHat v2.0.12 [47]. The numbers of reads matched to single genes were counted using the HTSeq v0.6.1 program [48]. The level of gene expression was estimated based on the FPKM (fragments per kilobase of transcript per million mapped reads) values. Genes were defined to be differentially expressed when they had a fold change of >2 or <0.5 as well as an adjusted *P*-value of <0.05. KEGG (Kyoto Encyclopedia of Genes and Genomes) pathway enrichment and Gene Ontology (GO) for differentially expressed genes (DEGs) were analyzed using the GOseq R package [49] and KOBAS software [50], respectively. The UPGMA method was selected to analyze hierarchical clustering.

Real-time PCR analysis was performed using Hieff^®^ qPCR SYBR Green Master Mix (Yeasen, stock number 11203ES03). The relative level of gene expression was normalized according to the value of the internal control gene *PpTEF2* [51]. The sequences of primers used are listed in Supplementary Data Table S6.

### GSK-J4 and 5-azacytidine treatments during peach leaf callus induction *in vitro*

DNA methylation alterations were achieved using DNA methyltransferase inhibitor 5-azacytidine [52] (Sigma–Aldrich, CAS number 320-67-2). To test the effect of 5-azacytidine on callus induction, leaves were incubated on CIM with 10 nM 5-azacytidine. Changes in histone modification patterns were achieved using the histone lysine demethylase inhibitor GSK-J4 [53]. To test the impact of GSK-J4 on callus induction, CIM was supplemented with 24 μM GSK-J4 (Targetmol, CAS number 1797983-09-5), in the micromolar concentration range commonly used in previous studies [54]. CIM containing DMSO was used as control. For these drug treatments and control, the same batches of fully expanded leaves were collected from the above-mentioned *in vitro* clonal propagation shoots, cut into small pieces and randomly used for callus induction at the same time. Three biological replicates were conducted for both 5-azacytidine and GSK-J4 treatments.

## Supplementary Material

Web_Material_uhac132

## Data Availability

The raw sequencing reads have been deposited in the Sequence Read Archive (SRA) database of NCBI under accession number PRJNA819103.
