# Supplementary material for: Epigenetic reprogramming of H3K27me3 and DNA methylation during leaf-to-callus transition in peach
Source: Hortic Res. 2022 Jun 3;9:uhac132. doi: 10.1093/hr/uhac132 (PMC9350832; doi:10.1093/hr/uhac132)
Supplement: Web_Material_uhac132 [file web_material_uhac132.zip › Supplementary Figures.docx]

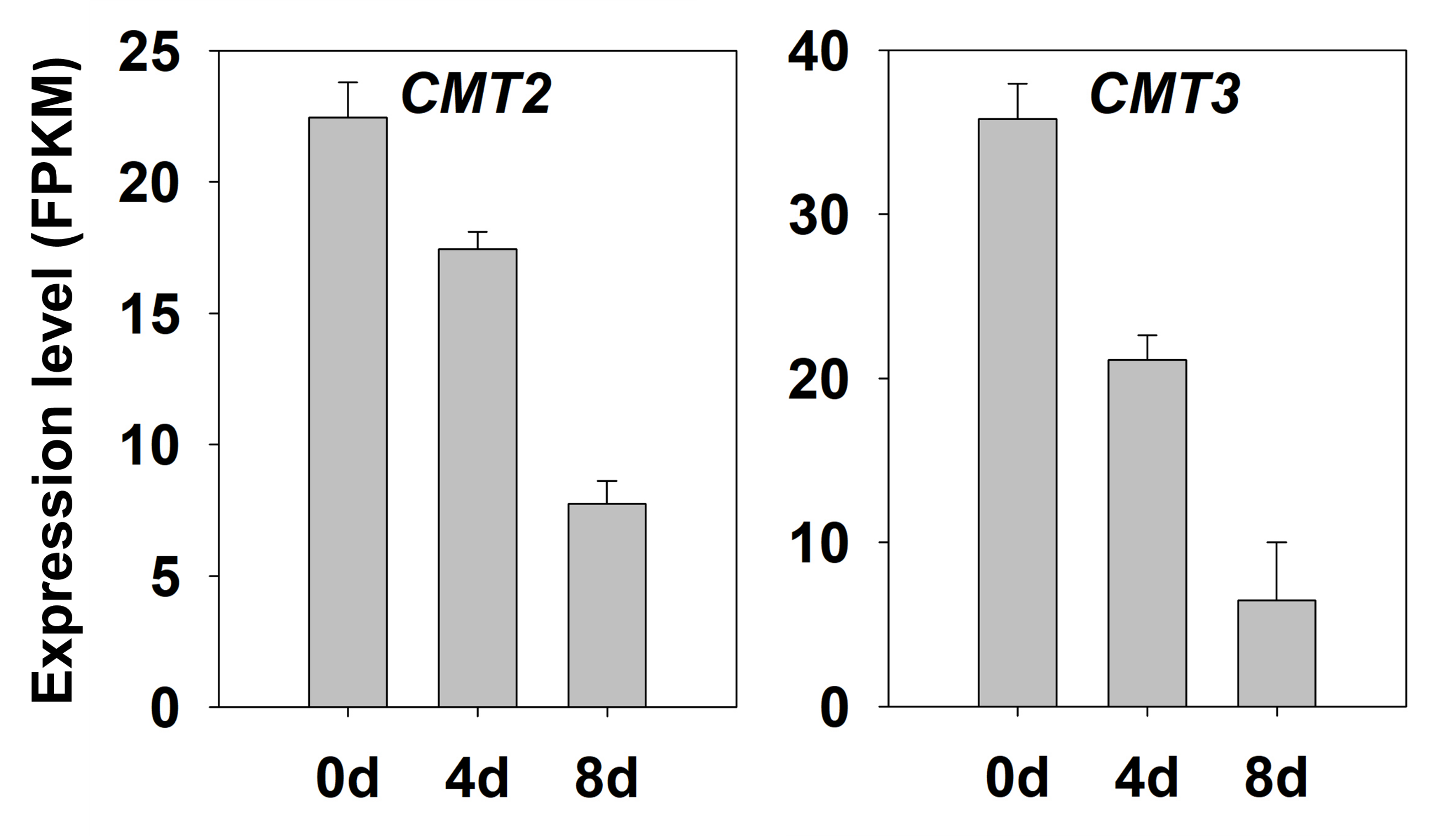


**Fig. S1 FPKM value of genes encoding homologs of DNA methyltransferase of leaves on CIM for 0, 4 and 8 days.**


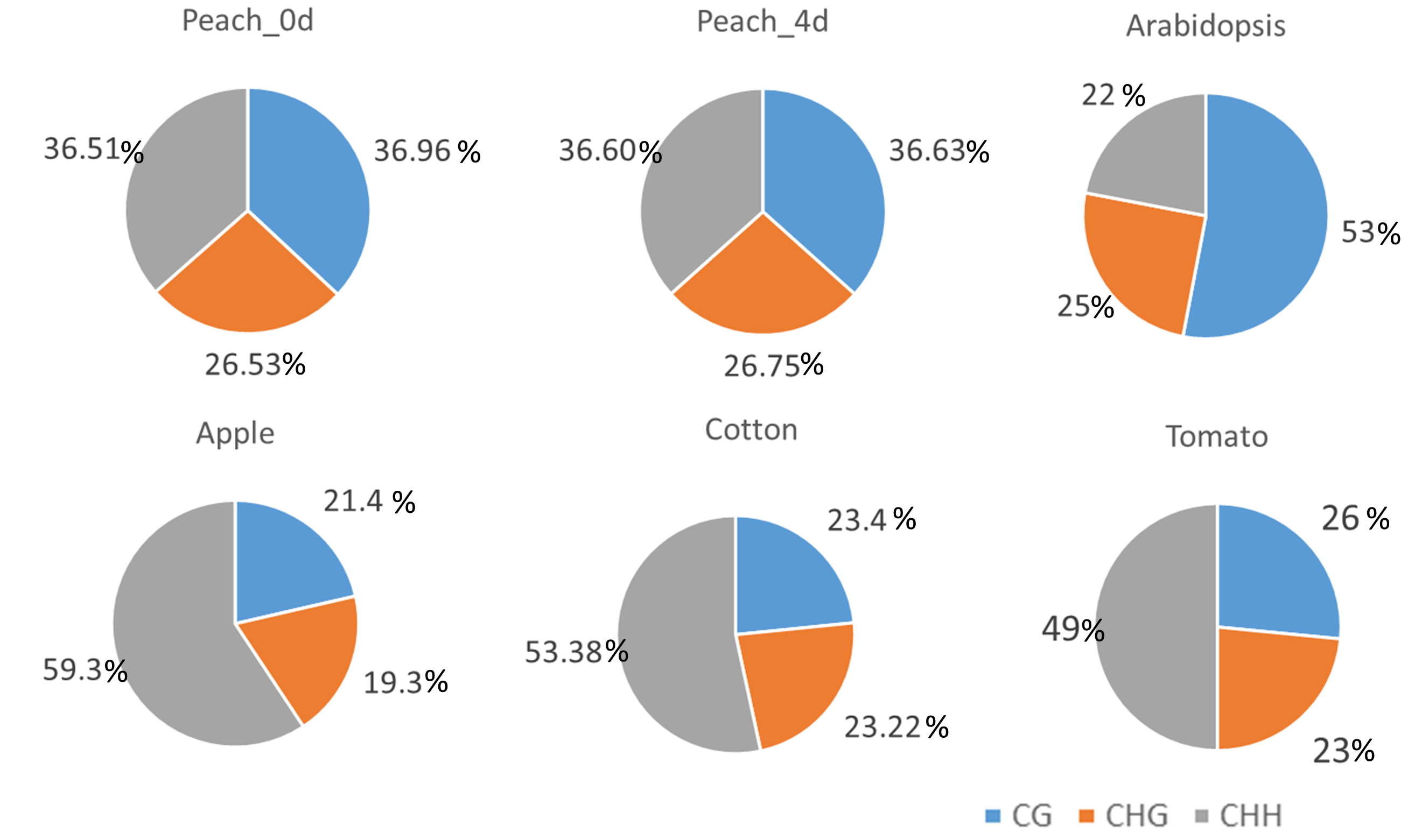


**Fig. S2 Relative proportions of mCs in the three C methylation types (CG, CHG and CHH) in peach, Arabidopsis, tomato and apple.**


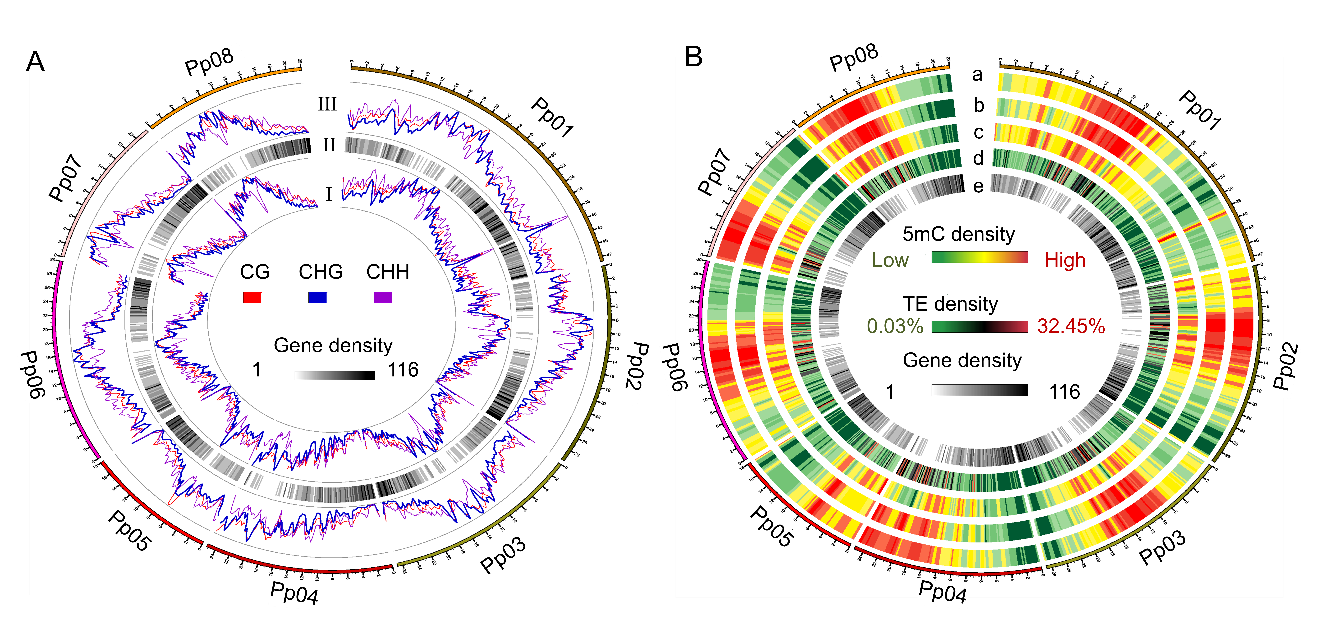


**Fig. S3 The peach epigenome of leaf explants in 0-day samples.** (A) Circos plot showing the level (I) and density (III) of mCs in the CG, CHG, and CHH contexts and gene density (II) in each peach chromosome. For the line graph of the level and density of 5-mCs, the CG, CHG, and CHH sequence types are displayed as red, blue, and purple lines, respectively. (B) Cicros plots of peach chromosomes. Track order: a, density plot of mC in CG context; b, density plot of mC in CHG context; c, density plot of mC in CHH context; d, density of transposable elements (TEs); e, gene density of each chromosome.


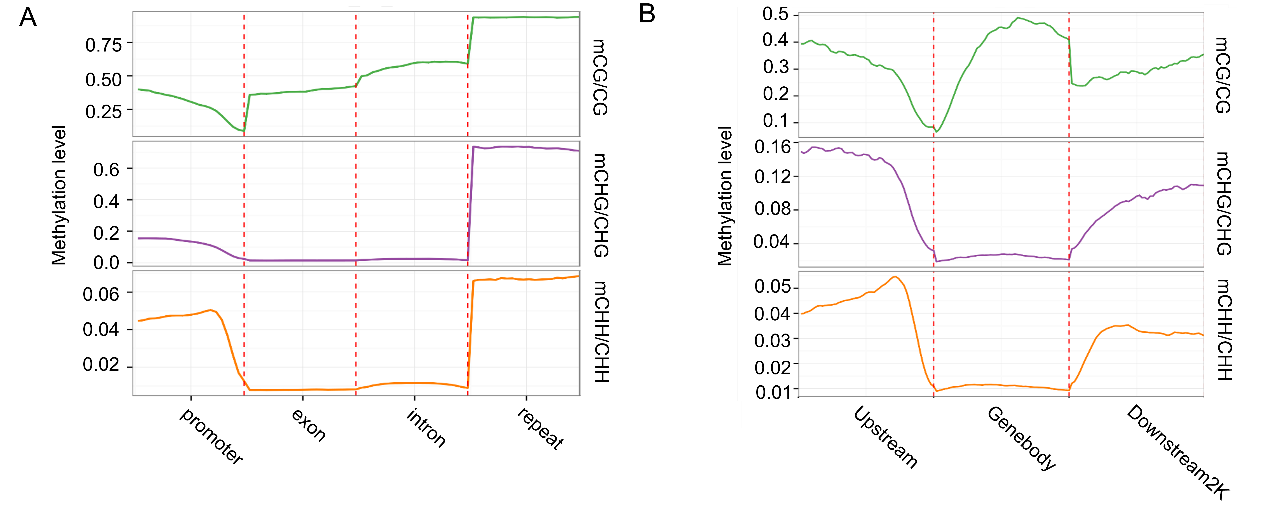


**Fig. S4 Profile of DNA methylation in genic regions.** (A) Distribution of DNA methylation levels within gene functional regions. The x and y-axes indicate gene functional domains and methylation levels, respectively. (B) Distribution of DNA methylation levels within gene-body domains and 2-kb upstream and downstream regions (TSS, transcriptional start site; TES, transcriptional end site). The x and y-axes indicate gene functional domains and methylation levels, respectively.


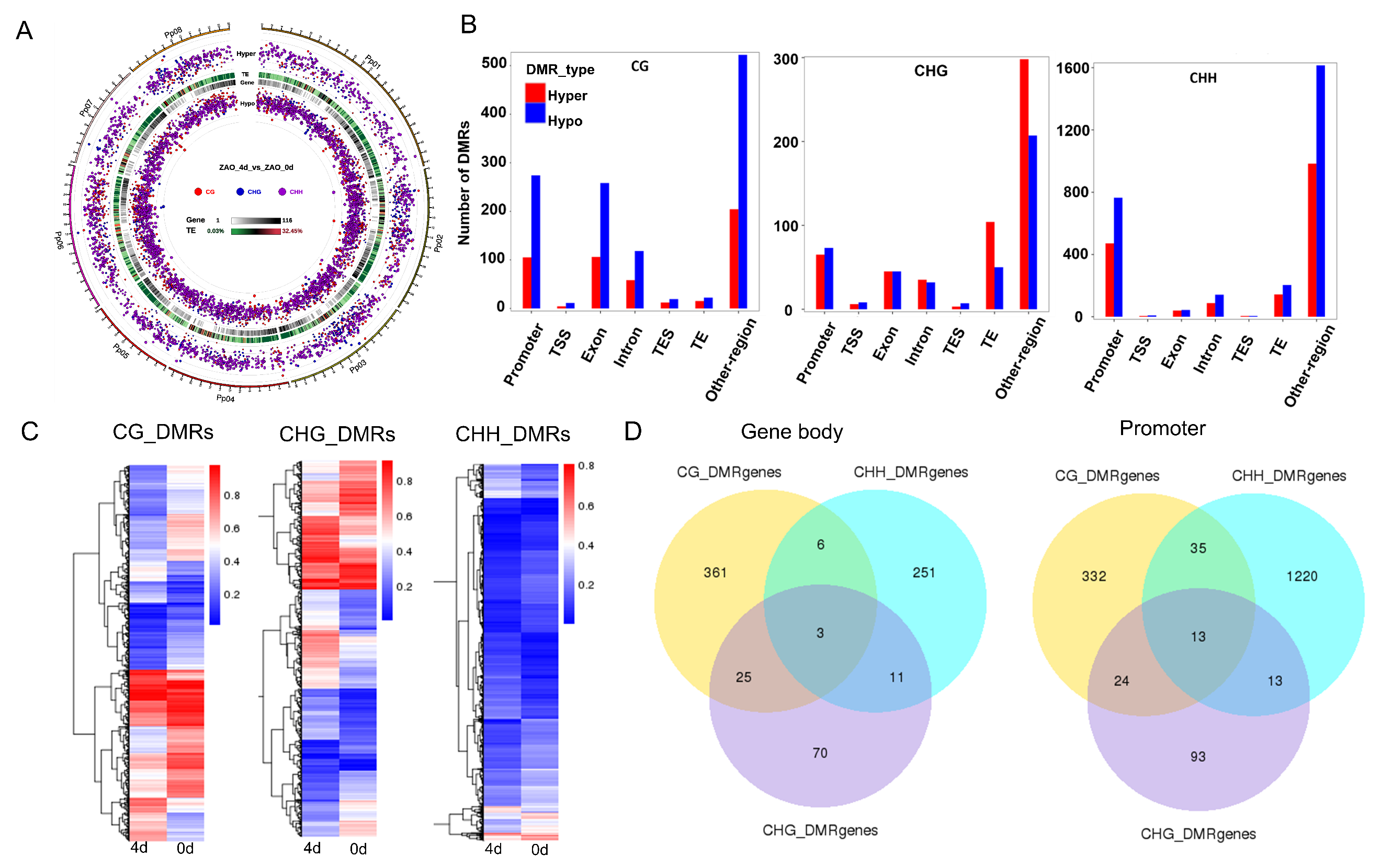


**Fig. S5 Summary of differentially methylated regions (DMRs) between 0 d and 4 d samples**. (A) Distribution of DMRs, transposable element and gene density on each chromosome of peach, a, hyper; b, TE; c, gene; d, hypo. (B) Numbers of CG, CHG, and CHH DMRs within gene regions. TSS, transcriptional start site; TES, transcriptional end site. (C) Heatmap of methylation levels of DMRs in three methylation context (CG, CHG and CHH). (D) Venn diagrams of predicted genes related to DMRs (CG, CHG and CHH). “Genebody” and “Promoter” indicated DNA methylation in gene body and promoter regions, respectively.


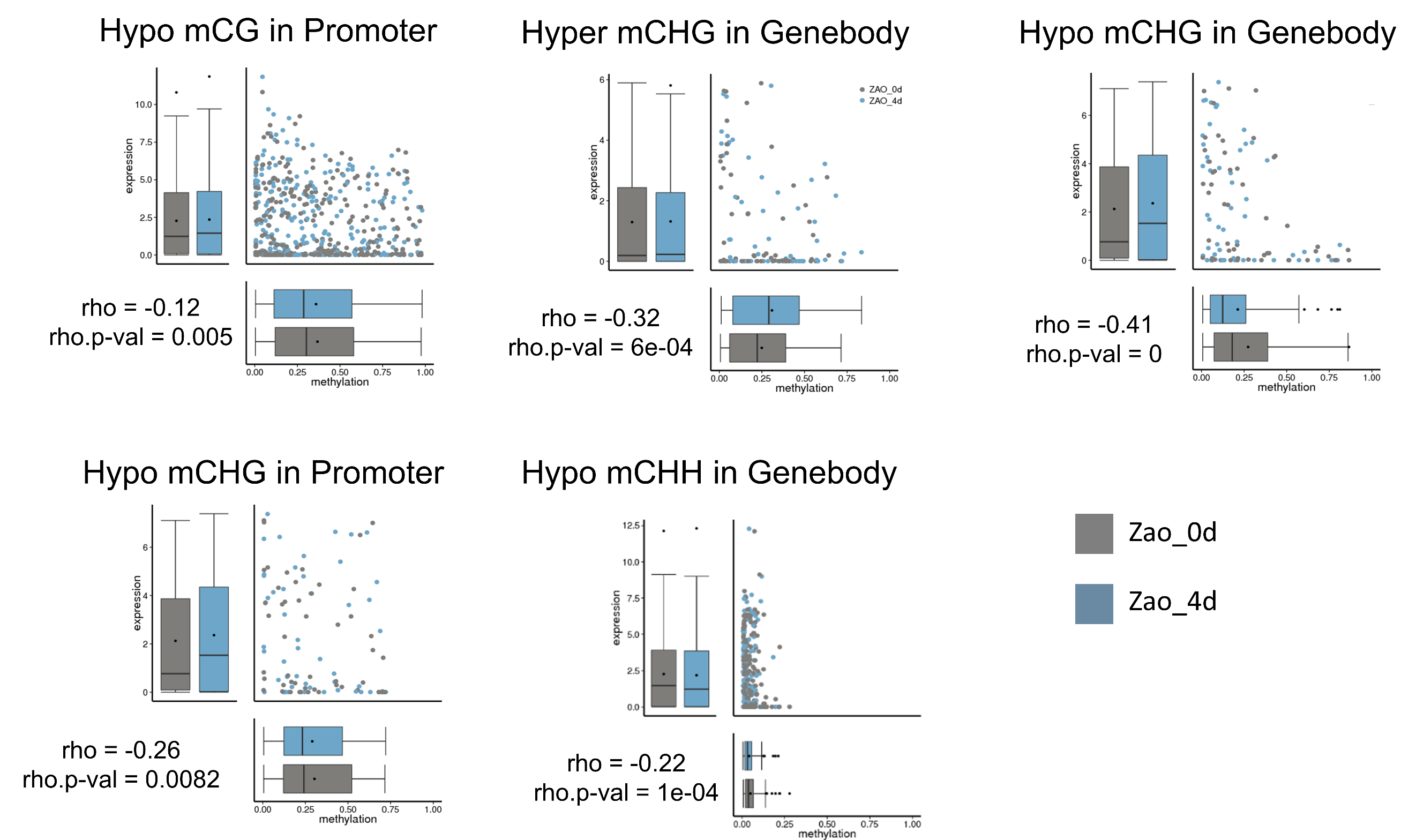


**Fig. S6 The correlation between DNA methylation levels and gene expression levels in DMRs-anchored genes.** Upper right, scatter plot of DMR-related gene expression vs. methylation level; bottom left, comparison and correlation statistics; bottom right, box plot of DMR-related gene methylation level.


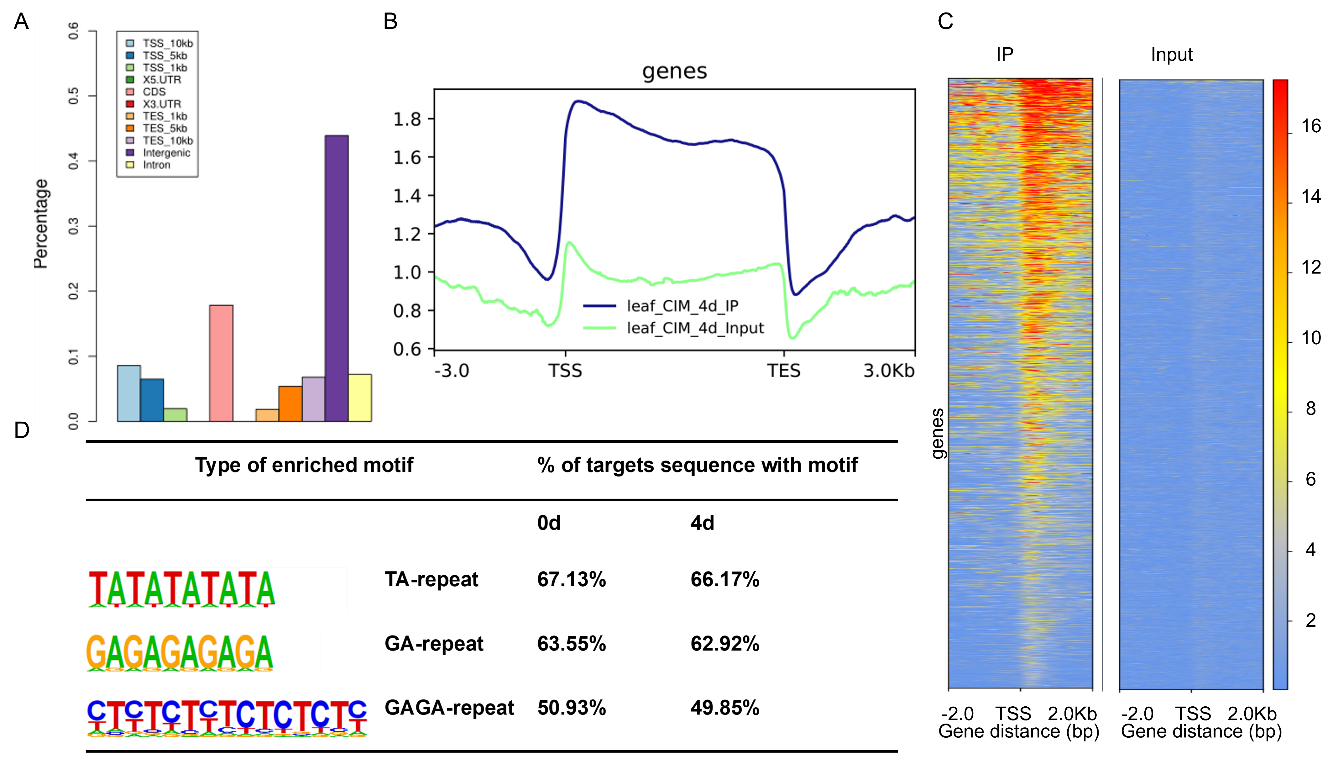


**Fig. S7 The characteristics of H3K27me3 in peach.** (A) The genomic distribution of H3K27me3 peaks within different regions in peach. (B) The distribution of H3K27me3 along all the peach genes (from 3-kb upstream regions to 3-kb downstream regions). (C) Heatmap of H3K27me3 deposition. (D) Enriched motifs of H3K27me3 binding region in peach.


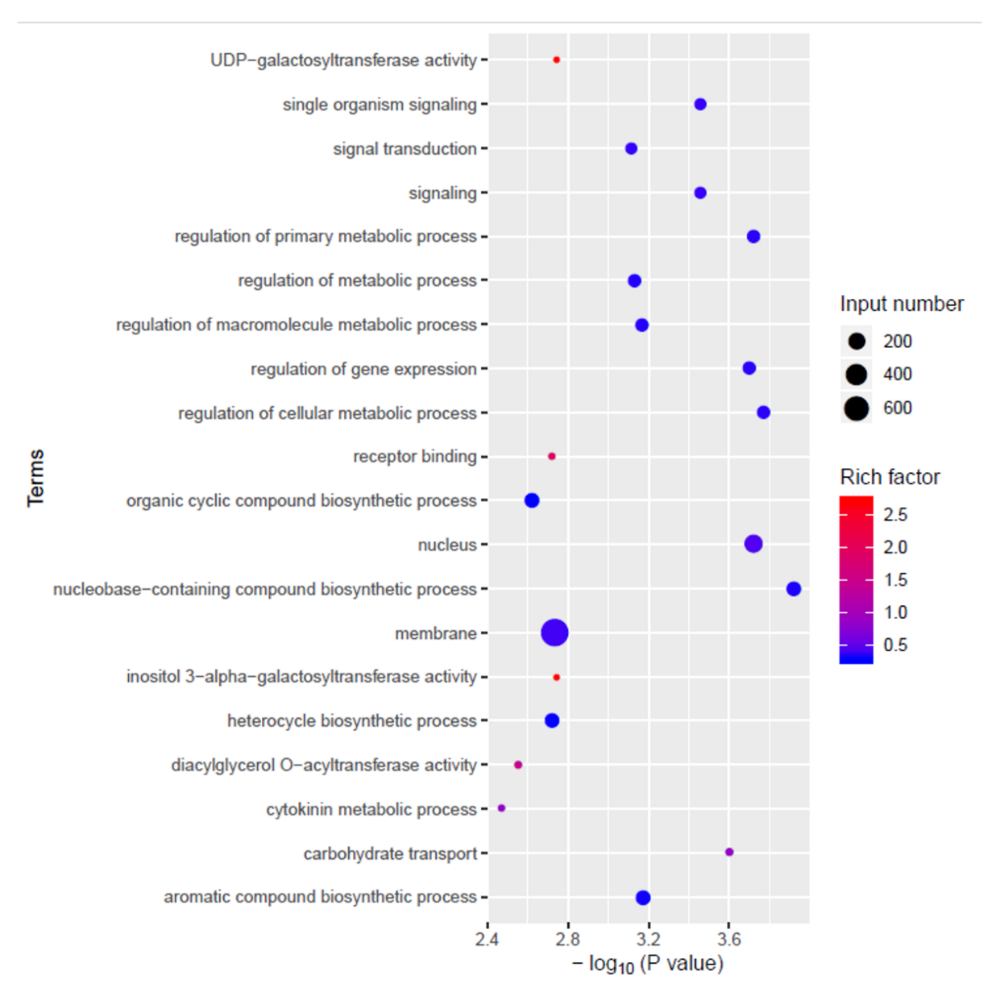


**Fig. S8 GO enrichment analysis of genes with decrease in H3K27me3 deposition during callus formation.**


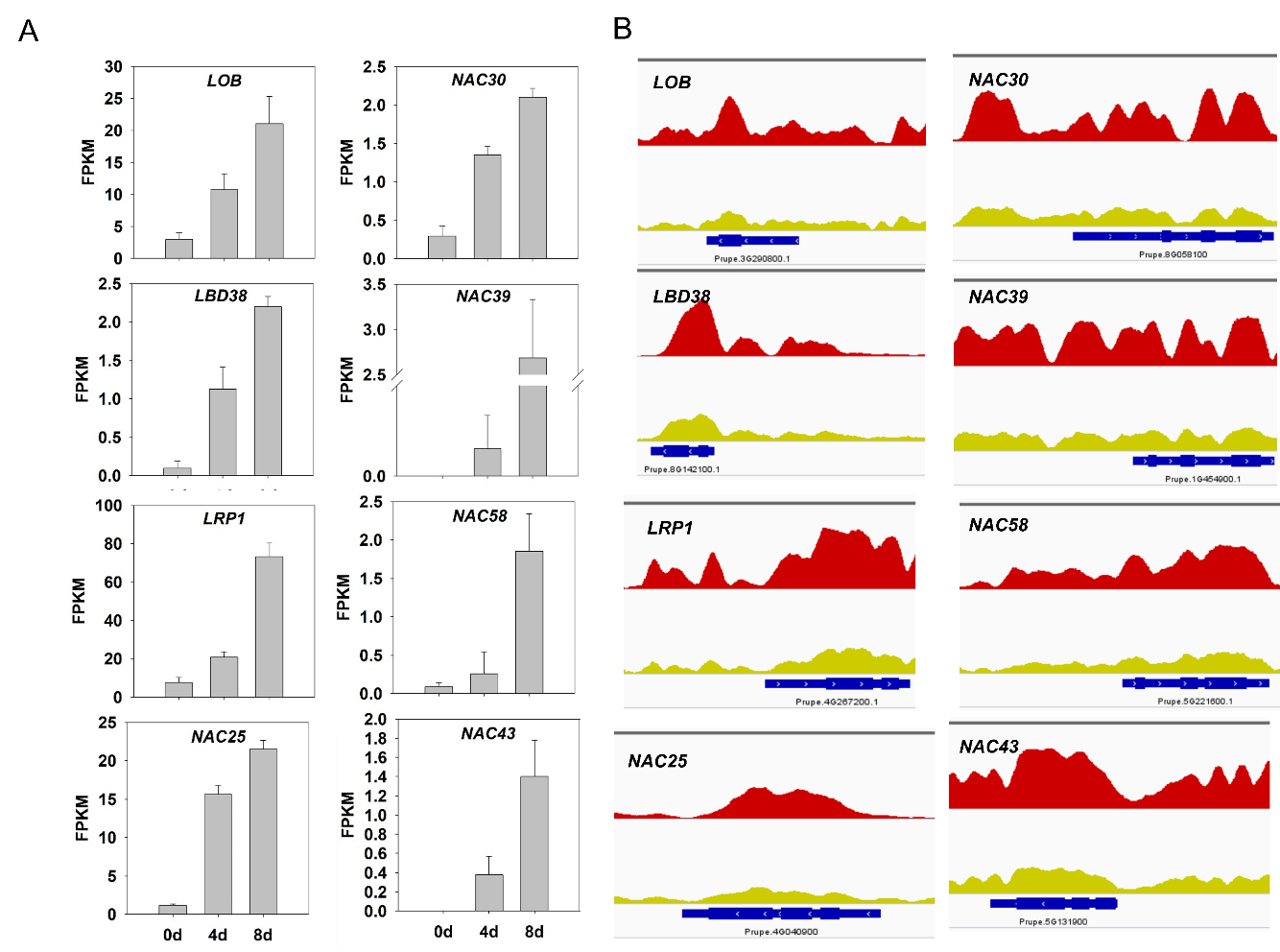


**Fig. S9 H3K27me3 hypomethylations occur at several transcription factors.** A, FPKM value showed increased expression levels of transcription factors. B, ChIP-seq results showed reduced H3K27me3 deposition.
